# Seltene Ursache der Heiserkeit – Falldarstellung und Kurzübersicht zur laryngealen Amyloidose

**DOI:** 10.1007/s00106-025-01681-6

**Published:** 2025-10-17

**Authors:** Hannah Schützenmeister, Rahel Schwotzer, Jakob Becker, Nathalie Nierobisch, Daniel Runggaldier, Jörg E. Bohlender

**Affiliations:** 1 Klinik für Otorhinolaryngologie, Head and Neck Surgery, Abt. für Phoniatrie und klinische Logopädie, Universitätsspital Zürich, Zürich, Schweiz; 2 Universität Zürich, Rämistrasse 71, 8006 Zürich, Schweiz; 3 Institut für Pathologie und Molekularpathologie, Universitätsspital Zürich, Zürich, Schweiz; 4 Klinik für Medizinische Onkologie und Hämatologie, Universitätsspital Zürich, Zürich, Schweiz; 5 Klinik für Neuroradiologie, Klinisches Neurozentrum, Universitätsspital Zürich, Zürich, Schweiz; 6 Institut für Diagnostische und Interventionelle Radiologie, Universitätsspital Zürich, Zürich, Schweiz; 7 Klinik für ORL, Universitätsspital Zürich, Frauenklinikstrasse 24, 8091 Zürich, Schweiz

**Keywords:** Mikrolaryngoskopie, Dysphonie, Stimmbelastung, Laryngeale Raumforderung, GRBAS-Skala, Microlaryngoscopy, Dysphonia, Vocal strain, Tumor-like laryngeal lesion, GRBAS scale

## Abstract

In dieser Fallübersicht wird ein Fall einer lokalisierten, laryngealen Immunglobulin-Leichtketten(AL)-Amyloidose beschrieben. Der Larynx ist einer der häufigsten Manifestationsorte dieser lokalisierten Form der Erkrankung. Die Amyloidose führt zu benignen, tumorartigen Läsionen, welche durch die Ablagerung nicht lösbarer Proteinaggregate, so genannter Fibrillen, entstehen. Im vorliegenden Fall präsentierte sich eine Patientin mit einer Larynxamyloidose und dem Leitsymptom Heiserkeit. Die häufigste therapeutische Maßnahme ist die chirurgische Abtragung des raumfordernden Gewebes, aber auch andere therapeutische Möglichkeiten existieren je nach Ausprägung und Verlauf, die wir ebenfalls in dieser Übersichtsarbeit zusammenfassen möchten.

## Abstract (en)

This case report describes a patient with localized laryngeal immunoglobulin light chain (AL) amyloidosis. The larynx is one of the most common sites of manifestation of localized amyloidosis. Amyloidosis leads to benign, tumor-like lesions caused by the deposition of insoluble protein aggregates, so-called fibrils. The current patient presented with the main symptom of hoarseness. Amyloidosis is most commonly treated by surgical resection of the mass. However, depending on the extent and course of disease, other therapeutic approaches are also feasible, which are discussed in this review.

## Anamnese

Die Vorstellung der 44-jährigen Patientin erfolgte aufgrund einer seit etwa einem halben Jahr bestehenden und im Verlauf deutlich zunehmenden Heiserkeit, die sich vor allem bei Stimmbelastung manifestierte. Weitere Symptome wie beispielsweise Dyspnoe, Reflux oder Schluckbeschwerden wurden verneint. Auch von einer B‑Symptomatik wurde nicht berichtet. Allergien oder andere Vorerkrankungen waren nicht bekannt. Die Patientin rauchte nicht und konsumierte nur gelegentlich Alkohol. Eine vorgängige kurzzeitige Therapie mit Esomeprazol 2 × 40 mg hatte die Symptomatik nicht gebessert.

## Befund

**Abb. 1:**
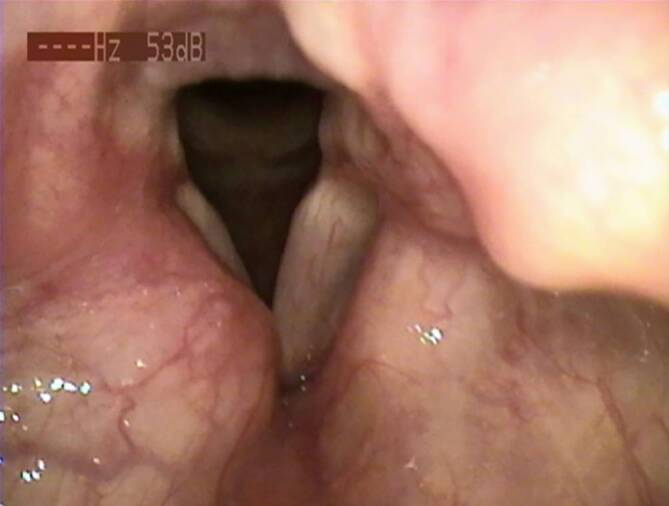
Präoperativer Befund in der fiberendoskopischen Untersuchung

In der Fiberendoskopie zeigte sich die rechte Taschenfalte tumorös verdickt bei ansonsten jedoch reizlosen Schleimhautverhältnissen insbesondere ohne Ulzerationen. Die rechte Stimmlippe selbst war aufgrund des Befundes nicht vollständig einsehbar. Die linke Stimmlippe präsentierte sich unauffällig (Abb. 1). Es bestand eine symmetrische Stimmlippenbeweglichkeit beim Wechsel von Phonations- zu Respirationsstellung und umgekehrt. Aufgrund der Heiserkeit war eine Stroboskopie nicht konklusiv durchführbar. Im Sonagramm zeigten sich unspezifisch verteilte Obertöne, der auditiv-perzeptive Befund wurde mit G2R1B2A0S1 beurteilt.

## Diagnostische Ergebnisse

**Abb. 2:**
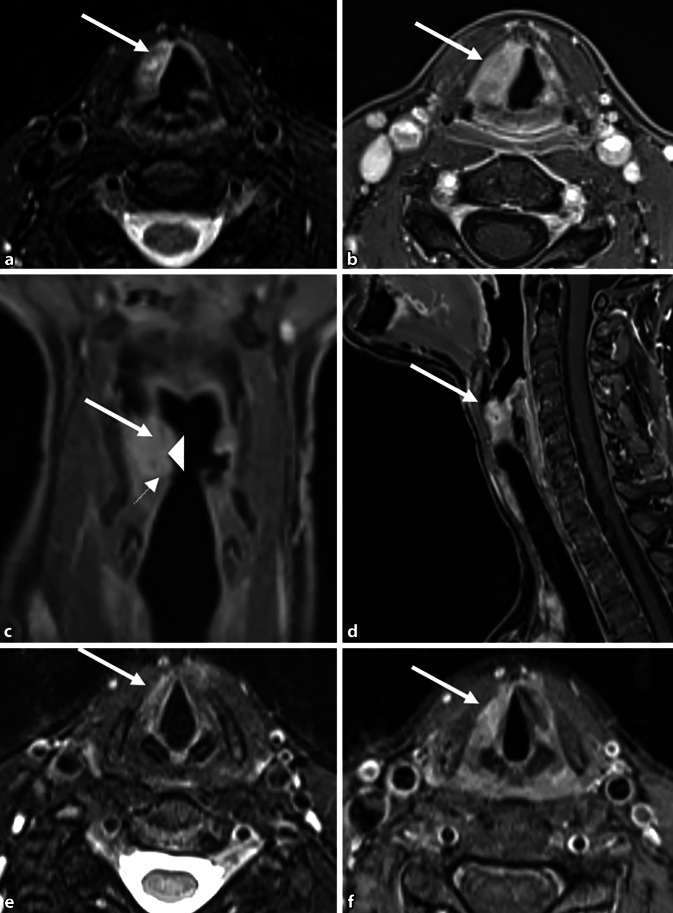
Hals-MRT mit Fokus auf den Larynx: **a** Axiale T2-gewichtete Fatsat-Aufnahmen, **b** axial, **c** koronar, **d** sagittal mit T1w-Bildern nach Gadolinium-Injektion. Rechts eine supraglottische T2w-hyperintense, kontrastmittelaufnehmende Masse in der Plica vestibularis (*Pfeil*) und in geringerem Maße in der Plica vocalis (*kleiner Pfeil*), die die rechte Taschenfalte (*Pfeilspitze*) überlagert und die linke aryepiglottische Falte etwas nach medial verschiebt. In der postoperativen Nachuntersuchung (**e** axiale T2w-Fatsat-, **f** axiale T1w-Bilder nach Gadolinium-Gabe) ist die Masse nach der Debulking-Operation kleiner und weniger kontrastverstärkt

Aufgrund des unklaren Prozesses wurde eine Magnetresonanztomographie (MRT) des Hals durchgeführt, bei dem sich nach Gadolinium-Injektion eine T2w-hyperintense, kontrastmittelaufnehmende Veränderung in der Plica vestibularis (Abb. 2b–d, Pfeil) zeigte. Zeichen für Hyperzellularität ergaben sich hierbei radiologisch nicht.

**Abb. 3:**
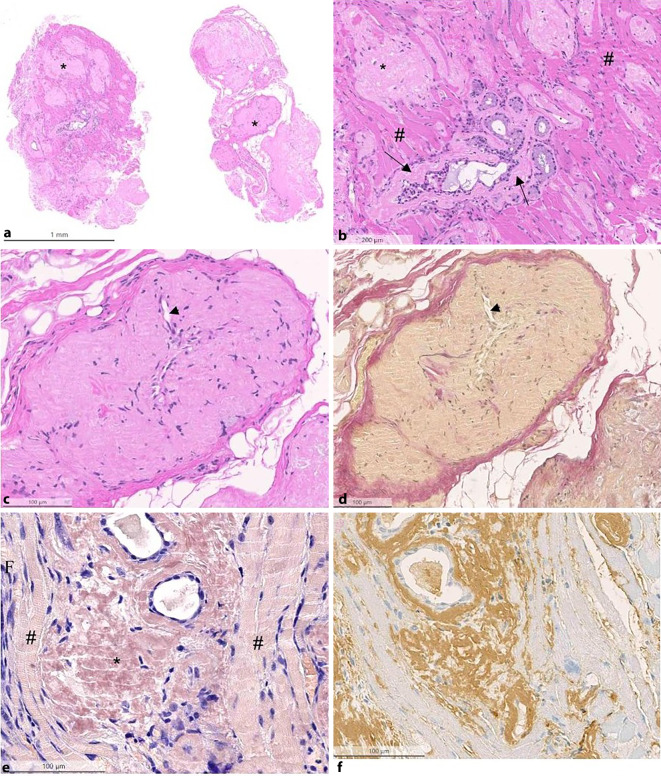
**a** Übersicht in der Hämatoxylin-Eosin(HE)-Färbung: Die kleinen Biopsien lassen bereits in der Übersicht rosafarbene knotige Veränderungen erkennen (*). **b** zeigt einen Ausschnitt aus der linken Biopsie aus (**a**). In der Skelettmuskulatur (*#*) sind die knotigen Ablagerungen gut erkennbar. Zusätzlich finden sich diese auch im Bereich der noch erkennbaren ortsständigen Drüsen (*schwarze Pfeile*). Die Amyloid-Ablagerungen weisen in der höheren Vergrößerung eine schollige Morphologie auf und sind zellarm. Nicht nur das Interstitium, sondern auch die Gefäße sind betroffen. Mittig kann noch das Lumen (*Pfeilköpfe*) erahnt werden (**c** und **d**). **d** In der Bindegewebefärbung (Elastika-van-Gieson-Färbung) sind kollagene Fasern rot, elastische Fasern schwarz. Gelb können Amyloidablagerungen oder auch Muskulatur sein. **e** Typischerweise ist Amyloid in der Kongorot-Färbung rötlich (*), hier in der Bildmitte. Links und rechts davon erkennt mal als Kontrast die Skelettmuskulatur, deren Querstreifung ebenfalls ersichtlich ist (*#*). **f** Negativität für Kappa-Leichtkette

Im Rahmen einer Mikrolaryngoskopie mit Biopsieentnahme zeigten sich im histopathologischen Befund mukoseröses Speicheldrüsengewebe sowie skelettmuskelhaltiges Weichgewebe mit diffusen Amyloidablagerungen (Abb. 3). Die HE-Färbung zeigte dabei knotige Veränderungen, wie in Abb. 3a, b dargestellt. In höherer Vergrößerung war die zellarme, schollige Morphologie zu erkennen. Nicht nur das Interstitium, sondern auch die Gefäße zeigten sich betroffen, wie in Abb. 3c, d erkennbar. In der Elastika-van-Gieson-Färbung konnten die Ablagerungen gelblich und in der Kongorot-Färbung hellrot gefärbt werden (Abb. 3e, Sternmarkierung), immunhistochemisch zeigte sich eine positive Reaktion für Leichtkette Lambda, sodass die Diagnose einer AL-Amyloidose Typ Leichtkette Lambda gestellt werden konnte.

Zum Ausschluss einer systemischen Beteiligung wurden verschiedene laboranalytische Untersuchungen durchgeführt. Das Blutbild war unauffällig. Serologisch fanden sich keine Hinweise auf das Vorliegen einer monoklonalen Gammopathie (MGUS), die eine zugrunde liegende Plasmazelldyskrasie bestätigt hätte. Es ließen sich auch in der klinischen Chemie keine indirekten Hinweise auf kardiale, renale oder hepatische Beteiligung finden. Eine Proteinurie war im Spoturin (in einer beliebigen Urinprobe) nicht nachweisbar. Die Echokardiographie war unauffällig. Eine Gastroskopie sowie eine Anoproktoskopie waren abgesehen von einem tubulären Low-Grade-Adenom des Colon ascendens regelrecht. Entsprechend konnte nach Abschluss dieser ausgedehnten Diagnostik eine lokalisierte Amyloidose des Typs Leichtkette Lambda bestätigt werden.

## Therapie und Verlauf

**Abb. 4:**
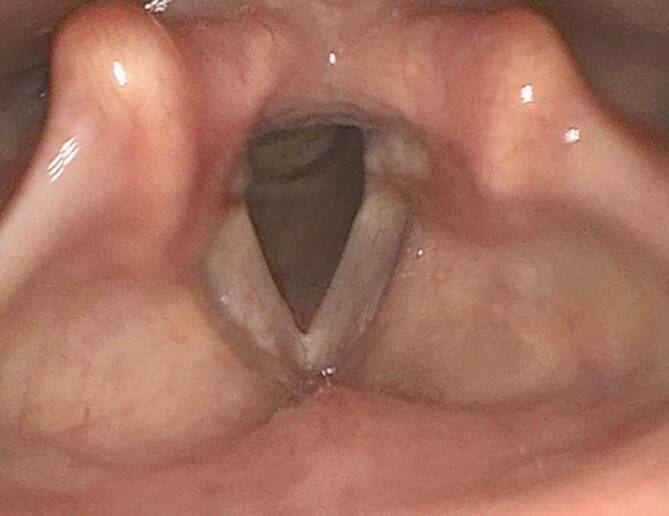
Endoskopische Aufnahme 5 Jahre postoperativ

Es erfolgte eine mikrochirurgische Abtragung im Rahmen einer erneuten Mikrolaryngoskopie: Zunächst wurde eine laterale Inzision in die rechte Taschenfalte gesetzt und der Befund sukzessive mikrochirurgisch vollständig herausgelöst und reseziert. In der postoperativen Laryngoskopie/Stroboskopie entwickelte sich im Resektionsgebiet an der Taschenfalte rechts initial ein pyogenes Granulom, das sich dann aber rasch von selbst regredient zeigte. Danach ergaben sich in unseren Kontrollendoskopien stets reizlose, regelrechte Verhältnisse mit bds. symmetrisch beweglichen Stimmlippen und regelrechter Randkantenverschieblichkeit (Abb. 4). Auditiv perzeptiv bestand stets eine unauffällige Stimme mit G0R0B0A0S0. Auch in den weiteren jährlichen Kontrollen ergaben sich stets unauffällige Befunde ohne Hinweis auf ein Lokalrezidiv oder Übergang in eine systemische Form.

## Diskussion

Eine Amyloidose ist durch fibrilläre Ablagerung von abnormal gefalteten Proteinen charakterisiert, zu der es meist im Interstitium oder auch in den Gefäßwänden kommt. Derartige fibrillöse Ablagerungen in ihrer typischen Beta-Faltblattstruktur werden als Amyloid bezeichnet. Es sind > 40 verschiedene Vorläuferproteine bekannt, die sich als Amyloid ablagern können [1, 2]. Die meisten Amyloidosen sind Systemerkrankungen und können grundsätzlich jedes Organ betreffen, wobei beispielsweise das Herz oder die Niere häufiger betroffen sind. Ätiologisch gibt es, je nach Vorläuferprotein, unterschiedliche Amyloidosen, welche zum einen hereditär und zum anderen erworben sein können [3]. Dazu gehören auch solche, welche im Rahmen anderer Grunderkrankungen auftreten (beispielsweise im Rahmen einer neoplastischen Plasmazellproliferation, chronische Infektionen u.v.m.) [1, 3].

Die Klassifikation der Erkrankung ist abhängig vom Vorläuferprotein. Der häufigste Typ ist die Transthyretinamyloidose (Vorläuferprotein Transthyretin), bei der man eine Wildtyp- (ATTRwt) und eine hereditäre Form (ATTRv) unterscheidet. Während bei der ATTRwt meist eine Herzinsuffizienz im Vordergrund steht, ist das klinische Bild der ATTRv uneinheitlicher und beinhaltet unter anderem auch Polyneuropathien [3]. Die Inzidenz der ATTRwt ist steigend, was v. a. auf die verbesserte Diagnostik und die verfügbaren Therapien zurückzuführen ist [4].

Als zweithäufigster Typ ist in der Schweiz die Immunglobulin-Leichtkettenamyloidose(AL)-Amyloidose zu nennen [5], welche bei der hier beschriebenen Patientin vorlag. Bei der systemischen Form der AL-Amyloidose stammt das amyloidogene Protein aus einer Plasma- oder reifen B‑Zellneoplasie im Knochenmark. Bei der lokalisierten Form ist der Produktionsort mutmaßlich lokal, eine monoklonale Gammopathie/Plasmazelldyskrasie liegt nicht vor.

Weitere Formen sind die entzündlich bedingte AA-Amyloidose (häufigste Form im globalen Süden) oder seltenere hereditäre Formen (Fibrinogen-Amyloidose, Lysozym-Amyloidose u. a.). Die AA-Amyloidose tritt sekundär bei Vorliegen langanhaltender Entzündungsprozesse auf, wie beispielsweise einer chronischen Infektion mit Mycobacterium tuberculosis, rheumatoider Arthritis oder auch chronisch-entzündlichen Darmerkrankungen. Es lässt sich eine Fehlfaltung und Aggregation des Proteins Serum-Amyloid A (SAA) nachweisen. Die Therapie dieser Form der Amyloidose zielt vor allem auf die Kontrolle der Grunderkrankung ab [3].

Lokalisierte Amyloidoseformen sind selten, typischerweise handelt es sich um lokalisierte AL-Amyloidosen. Man geht davon aus, dass der Produktions- und Ablagerungsort der Fibrillen identisch ist, d. h. eine Plasma- oder reife B‑Zelle die amyloidogene Leichtkette am Ablagerungsort produziert [1]. Amyloidablagerungen im Kopf-Hals-Bereich können im Sinne einer lokalen Schwellung auftreten. Sie finden sich jedoch auch bei vielen Patienten mit systemischer Amyloidose [6].

Beschriebene Manifestationsorte der lokalen Amyloidose im Kopf-Hals-Bereich sind Orbita, Nasopharynx, Lippen, Mundboden, Zunge, das urogenitale und das tracheobronchiale System [7]. Am häufigsten betroffen ist der Larynx. Dort finden sich die Amyloide wie in unserem Fall am häufigsten im Bereich der Supraglottis, z. B. in der Plica vestibularis [6, 8].

Patienten mit laryngealer Amyloidose stellen sich häufig mit Beschwerden wie Heiserkeit, Dyspnoe oder Stridor vor. Auch ein Globusgefühl oder Hustenreiz sind mögliche Symptome. Generell hängt die Ausprägung der Symptomatik stark von Lokalisation und Größe der Läsionen ab [7–10].

In der Diagnostik spielen neben der HNO-ärztlichen Untersuchung auch bildgebende Verfahren eine Rolle. Meist wird die laryngeale Amyloidose initial als tumorsuspekte Läsion in der direkten Laryngoskopie interpretiert [8]. Die Läsionen präsentieren sich häufig submukosal [11], teilweise auch mit zystischen Anteilen [7]. Die Amyloidmasse erscheint makroskopisch häufig gelblich-orange oder auch rötlich vom farblichen Aspekt ([10, 12, 13] Abb. 3e, Sternmarkierung). Bildgebende Verfahren wie CT und MRT stellen eine sinnvolle Ergänzung der Diagnostik dar, um das Ausmaß der Amyloidose zu bestimmen und mit den Symptomen der Patienten zu korrelieren. In der MRT ist die gut abgrenzbare, homogene, submuköse Läsion in der T2-Sequenz hyperintens sichtbar (Abb. 2a, Pfeilmarkierung). Eine CT könnte zusätzliche Informationen liefern, da hier in einigen Fällen Kalzifikationen nachgewiesen werden können [11]. Bei der vorliegenden Patientin wurde aufgrund von fehlender Konsequenz bei guter chirurgischer Resektabilität auf eine solche zusätzliche CT bewusst verzichtet. Eine definitive Diagnose kann dann aber nur durch histopathologische Aufarbeitung der Biopsie oder des Exzisionspräparats gesichert werden:

Konventionell-lichtmikroskopisch erkennt man in der HE-Färbung meist knotige, schollige, zellarme, eosinophile Ablagerungen (Abb. 3b, Rautenmarkierung). Amyloidablagerungen können ein unterschiedliches Ausmaß annehmen von nur sehr diskreten Befunden bis hin zu ausgeprägten tumorösen Gewebsläsionen. Besteht lichtmikroskopisch die Verdachtsdiagnose einer Amyloidose, so können zusätzliche Färbungen die Diagnose weiter erhärten. Typischerweise ist eine Amyloidose in der Elastika-van-Gieson-Färbung gelblich (Abb. 3d, Pfeilmarkierung) und in der Kongorot-Färbung, dem Goldstandard zur Anfärbung von Amyloiden, hellrot (Abb. 3e, Sternmarkierung). Eine Typisierung des Amyloids ist zwingend, um die korrekte Diagnose zustellen.

Bei Erstdiagnose einer lokalen Amyloidablagerung muss eine systemische Amyloidose ausgeschlossen werden [8, 9, 12, 14, 15]. In der aktuellen Leitlinie zur Diagnostik und Therapie der extrazerebralen Amyloidosen wird als Basisdiagnostik neben einer ausführlichen Anamnese und körperlichen Untersuchung/Statuserhebung auch eine umfassende Labordiagnostik empfohlen. Diese umfasst neben Funktionsparametern der Niere (Elektrolyte, Kreatinin, Harnstoff), Leber (AP, GGT, ASAT, ALAT, CHE, Albumin, INR) und Schilddrüse (TSH, fT3, fT4) auch allgemeine Entzündungsparameter (BSG, CRP), sowie kardiale Biomarker (NT-proBNP und Troponin) und den Ausschluss einer Proteinurie. Ein Gammopathiescreening (Immunfixation im Serum und Urin, freie Leichtketten im Serum) gehört zur Basisdiagnostik ebenfalls dazu. Zudem wird eine Röntgenaufnahme des Thorax und ein Echokardiogramm empfohlen [16]. Bei bestätigter MGUS sollte eine Biopsie aus dem Knochenmark entnommen werden [11]. In unserem Fall erfolgte eine umfassende Abklärung in den Spezialsprechstunden für Kollagenosen und Vaskulitiden der Klinik für Rheumatologie sowie der Amyloidosespezialsprechstunde der Klinik für Medizinische Onkologie und Hämatologie des Universitätsspitals Zürich.

Die Therapiemöglichkeiten der lokalisierten Amyloidose umfassen die chirurgische Resektion, die adjuvante Radiotherapie sowie das abwartende Beobachten [9]. Eine medikamentöse Therapie ist bisher nicht etabliert. Die mikrolaryngoskopische Resektion mit herkömmlichen mikrochirurgischen Instrumenten, einem Microdebrider oder CO2-Laser stellen die am häufigsten durchgeführten Therapiemethoden dar [15, 17]. Da es sich bei der lokalisierten Amyloidose um einen langsam fortschreitenden Prozess handelt, besteht das Hauptziel des operativen Eingriffs in der Reduktion der Symptomlast der Patienten und nicht primär in der vollständigen Exzision der Läsion [9]. Ein erneutes Auftreten von Symptomen nach der chirurgischen Resektion ist häufig [8, 15]. In unserem Fall zeigt sich die Patientin jedoch auch fünf Jahre postoperativ nach chirurgischer Resektion mit anschließender logopädischer Kurzintervention beschwerdefrei ohne Hinweis auf ein Rezidiv. In vielen in der Literatur beschriebenen Fällen war jedoch eine wiederholte chirurgische Resektion erforderlich. Zudem ist bei einigen dieser beschriebenen Patienten eine postoperative Radiotherapie durchgeführt worden, der man eine Reduktion der Rezidivwahrscheinlichkeit zuschreibt [18]. Laut Hazenberg et al. treten erneute Amyloidablagerungen meist in den ersten sieben Jahren nach der initialen Diagnosestellung und Therapie auf, weshalb die Autoren diesen Zeitraum für regelmäßige Follow-up-Kontrollen empfehlen [15]. Risikofaktoren für ein erneutes Auftreten von Amyloidablagerungen sind ein jüngeres Alter bei Auftreten der Beschwerden und ein großer Zeitverzug zwischen Diagnose und Resektion. Zudem wurde ein subglottisches Auftreten der Läsionen als unabhängiger Risikofaktor identifiziert [19].

Die in diesem Fall beschriebene Patientin befindet sich derzeit im fünften postoperativen Jahr ohne Hinweis auf ein Rezidiv. Nachkontrollen erfolgen dabei im jährlichen Intervall. Aufgrund des guten Verlaufs haben wir bei unserem Fall auf eine adjuvante Behandlung mit einer Radiotherapie bewusst verzichtet.

## Fazit für die Praxis

- Bei submukösen tumorösen Läsionen im Larynx ist differenzialdiagnostisch auch an eine lokalisierte laryngeale Amyloidose zu denken.
- Eine zeitnahe laryngologische Beurteilung mit frühzeitiger Biopsie bzw., wenn möglich, mit phonochirurgischer Exzision ist anzustreben.
- Das Vorliegen einer systemischen Manifestation sollte bei Erstdiagnose einer laryngealen Amyloidose ausgeschlossen werden.
- Hierfür ist die Anbindung an eine auf Amyloidose spezialisierte Abteilung zur Durchführung der weiteren Diagnostik sinnvoll.
- Ein systematisches Follow-up über einen Zeitraum von mindestens sieben Jahren wird empfohlen.
